# ‘Go fish’: Conceptualising the challenges of engaging national web archives for digital research

**DOI:** 10.1007/s42803-021-00032-5

**Published:** 2021-04-27

**Authors:** Jessica Ogden, Emily Maemura

**Affiliations:** 1 University of Bristol, Bristol, UK; 2 University of Toronto, Toronto, Canada

**Keywords:** National web archives, Digital research methods, Scholarly primitives, Digital curation

## Abstract

Our work considers the sociotechnical and organisational constraints of web archiving in order to understand how these factors and contingencies influence research engagement with national web collections. In this article, we compare and contrast our experiences of undertaking web archival research at two national web archives: the UK Web Archive located at the British Library and the Netarchive at the Royal Danish Library. Based on personal interactions with the collections, interviews with library staff and observations of web archiving activities, we invoke three conceptual devices (*orientating, auditing* and *constructing*) to describe common research practices and associated challenges in the context of each national web archive. Through this framework we centre the early stages of the research process that are often only given cursory attention in methodological descriptions of web archival research, to discuss the epistemological entanglements of researcher practices, instruments, tools and methods that create the conditions of possibility for new knowledge and scholarship in this space. In this analysis, we highlight the significant time and energy required on the part of researchers to begin using national web archives, as well as the value of engaging with the curatorial infrastructure that enables web archiving in practice. Focusing an analysis on these research infrastructures facilitates a discussion of how these web archival interfaces both enable and foreclose on particular forms of researcher engagement with the past Web and in turn contributes to critical ongoing debates surrounding the opportunities and constraints of digital sources, methodologies and claims within the Digital Humanities.

## Introduction

Web archives have become a key source for web-based research and recent anthologies provide examples of their use in a wide range of disciplines, including Digital Humanities research (Brügger, 2017; Brügger & Laursen, 2019; Brügger & Schroeder, 2017; Milligan, 2019). Much of this work has drawn on large-scale collections, with a particular focus on the use of national web archives. While this scholarship demonstrates how web archives afford new opportunities for digital research, they have also identified a persistent ‘sociotechnical gap’ (Ackerman, 2000) between the needs of researchers and the affordances of current web archival research infrastructures - a problem neatly summarised by Lepore:

*“You can do something more like keyword searching in smaller subject collections, but nothing like Google searching (there is no relevance ranking, for instance), because the tools for doing anything meaningful with Web archives are years behind the tools for creating those archives. Doing research in a paper archive is to doing research in a Web archive as going to a fish market is to being thrown in the middle of an ocean; the only thing they have in common is that both involve fish”* (Lepore, 2015)*.*

Though Lepore notes a lack of search and discovery tools, in recent years strides have been made to further the academic use of web archives through the creation of programmes and open source software, including faceted search interfaces for large-scale collections like ArchiveSpark^1^ and those developed by the Archives Unleashed Project.^2^ Many web archiving research tools to date have focused on addressing what could be characterised as the ‘technical challenges’ of engaging with these (often) large-scale archival collections, with emphasis placed on developing ‘something like Google searching’ as a way to facilitate search and scholarly use. However, despite a recent upswing in international research initiatives focused on these analysis tools, many significant challenges remain.

These challenges have been collated elsewhere, including Maemura (2018) who synthesises common problems, such as defining a corpus of study within web archival collections, evaluating the suitability of collected materials and considering the implications of ethics and consent. Most recently, Vlassenroot et al. (2019) review the landscape of web archiving activities within the European context, and summarise four ‘important considerations’ for digital scholarship, including: how and why selection activities take place, the access conditions and legal frameworks that govern web archives, and the ‘high level of technical knowledge’ required to contextualise and make use of collections data. Whilst Vlassenroot et al. connect some of the challenges of researcher use with the very nature of the archival process, little work has empirically and comparatively addressed how researcher engagement is intricately connected to the complex processes of web archival scoping, collection and curation, in practice. Issues associated with researchers’ desires to evaluate the provenance of source materials (Maemura et al., 2018) and the complex sociotechnical assemblage of web archival infrastructures - including the diverse institutional access restrictions and protocols (such as those defined by legal deposit), limited tools and interfaces for engaging archived web materials at multiple scales (Lin et al., 2014), and the often obscured or invisible labour of human and automated curatorial interventions (Ben-David & Amram, 2018; Ogden et al., 2017) - all combine to constrain the ways researchers come to know web archives as sources for research.

Given this known set of constraints, we explore possible ways for engaging large scale national web archives at the earliest stages of research and how, in turn, national web archival infrastructures themselves shape the aims, questions and the possibilities of research. Our work considers the sociotechnical and organisational constraints of web archiving in order to identify and understand how these factors and contingencies influence subsequent research engagement with collections. Drawing from our experiences of two separate research projects, we investigate how the scholarly potential of the archived Web collides with the very real barriers researchers encounter when attempting to use these increasingly critical resources. In this article, we compare and contrast our experiences of undertaking web archival research at two national web archives: the UK Web Archive (UKWA) based at the British Library and the Netarchive at the Royal Danish Library. Our personal interactions with the collections are supplemented by interviews with library staff and observations of the everyday practices of web archivists, with a view towards understanding the similar yet different circumstances that shape researcher engagement in web archives.

As part of this work, we develop a framework to conceptualise the fundamental scholarly activities which researchers must engage with at the early stages of research in the context of national web archives. These conceptual devices (*orientating, auditing* and *constructing*) elucidate common practices but also their associated challenges in the context of each national web archive. We align our discussion along several dimensions, including: the legal mandates for collection; the ontological decisions and definitions that drive practices; the affordances and path dependencies of tools; the everyday infrastructural maintenance and labour involved in web archiving; and the ways in which all of the above defines and constructs the various interfaces through which web archives are accessed by researchers. Through the contribution of our conceptual framework and discussion, the article points to potential pitfalls and opportunities for future interventions aimed at encouraging the use of web archives in digital research.

## Outline of cases

The empirical work that informs this article stems from the authors’ separate studies at: the UK Web Archive (UKWA), based in the British Library; and the Netarchive, based at the Royal Danish Library. A brief description of both studies is provided here to contextualise this research, with further details provided in the discussion of each component of the conceptual framework.

### The UK Web Archive

The UKWA is a consortium of six legal deposit libraries who are collectively responsible for gathering, maintaining and providing access points to archived websites in the UK domain.^3^ To facilitate greater use of these web archives, the British Library (BL) and the UKWA have collaborated on several projects over the years (e.g. including the Big UK Domain Data for the Arts and Humanities [BUDDAH] project, and others) that have focused on building datasets, tools and relationships with external researchers across the arts and humanities, social sciences and computer sciences. These projects demonstrate both the opportunities and continued challenges for researchers grappling with how to meaningfully interpret search results and interfaces; issues of (in)completeness in web archives; and the processes of translating and integrating research practices into emerging theoretical and methodological frameworks for digital scholarship (Cowls, 2017; Winters, 2017).

The study that informs this article took place in the context of a three-month research placement at the BL in 2018, funded by the UKRI/ESRC National Centre for Research Methods. The aims of the placement were to identify social science research opportunities using UKWA collections, with a self-directed research focus on the UKWA News Collection. From July–September 2018, the researcher was based at the BL within the UKWA team, with a substantive focus on developing a case study using the Hyperlocal News collection. During this time, and in order to contextualise and understand the work surrounding the collection practices associated with the archives, informal discussions were undertaken with BL and UKWA staff in a variety of roles. These conversations included UKWA curators, engagement officers and technical support; BL staff responsible for various digital collections and digital scholarship engagement, as well as researchers based at the BL engaged in building web archival collections. The study included the production of a historical review of UKWA projects with researchers (with a focus on the specific opportunities and challenges presented by the UKWA for researchers), as well as created the foundations for a substantive case study on the discursive representations of immigration and migration in UK News Media. Overall, in addition to this substantive research focus, the placement was led by the question, ‘what do researchers need to know about web archive collections’, with a view towards informing future UKWA policy and development.

### The Danish Netarchive

The Netarchive is a legal deposit collection representing ‘the Danish Part of the internet’, with ongoing, extensive crawling of the Danish web domain since July 2005. The collection began as a collaboration between the Royal Library in Copenhagen, and the State and University Library in Aarhus. While these were initially two separate institutions, an organisational merger occurred in late 2017, and the two locations are now collectively referred to as ‘The Royal Library’. The collection has also developed in close connection with researchers from the Centre for Internet Studies at Aarhus University, who collaborated on an early pilot project in 2001, and have had a seat on the Netarchive’s Advisory Board since 2005. Overall, researcher engagement with the collection has largely centred on doctoral student uses, as well as recent work facilitated by Aarhus University’s NetLab, which has a mandate to provide support, training and outreach to researchers in Denmark studying internet materials. Most recently, the Royal Library has collaborated with NetLab’s researchers on a pilot project testing the library’s National Heritage Computing Cluster, a purpose-built high-performance computing cluster for data analysis of their digital cultural heritage collections.

The study that informs this article took place in the context of a PhD research project addressing provenance and decision-making for web archives collections. On-site fieldwork included observations and interviews of library curators, developers, researchers and managers over three months (February to May 2018). Findings additionally draw upon experiences and impressions of the collection as a research user, and through desk research studying the publicly available documentation from the Netarchive’s website, public meeting notes and software documentation. This study of the Netarchive was focused more on understanding collection and curation processes, and less on answering a specific research question using the Netarchive as a source. Direct engagement with the collection was largely exploratory and limited by Danish language/culture barriers, as well as access restrictions to data (discussed further below). Previous experiences with Archives Unleashed datathons and smaller Archive-It collections from Canadian academic libraries influenced this work, and initial explorations attempted to translate similar methods to the context of working with the Netarchive. Subsequent search strategies were guided by topics and examples that came up in discussion and interviews, including attempts to re-trace the steps of past research projects that used the Netarchive.

## Developing a conceptual framework

In an effort to inform future research in this space, we develop a conceptual framework to structure the challenges that researchers must contend with during the early stages of using national web archives. Broadly, there has been considerable attention paid to understanding humanities and social science ‘information work’, or the activities that underpin common scholarly research practices. Within the humanities, this work is best embodied by efforts to identify so-called ‘scholarly primitives’ (Unsworth, 2000) - or the ‘basic functions common to scholarly activity across [humanities] disciplines’ (Blanke & Hedges, 2013 p. 655) - and translate them into specific infrastructural or technology interventions that facilitate these practices within digital research. Unsworth initially describes several primitives (Discovering, Annotating, Comparing, Referring), which have been expanded and refined by others investigating user experience design and digital tool development (Blanke & Hedges, 2013; Trace & Karadkar, 2017). Other work in web archives has similarly focused on developing ‘task models’ or ‘process models’ for big data tools, most recently with the FEAV model (Filter, Extract, Aggregate and Visualize), which was developed in conjunction with the Archives Unleashed Toolkit (Jackson et al., 2016; Lin et al., 2017; Ruest et al., 2020).

Inspired by (yet in contrast to) the approach of scholarly primitives and the above examples, we present a conceptual framework that maps more specifically to the earliest stages of web archival research practices. Reflecting on our own experiences in the early stages of the research process, we emphasise activities that are often only given cursory attention in methodological descriptions of web archival research. Here we embrace the lessons that can be learned through ‘specific accounts of what actually happened to people doing hands-on work’ (Hargittai & Sandvig, 2015, p.1) and use these narratives to discuss the epistemological entanglements of researcher practices, instruments, tools and methods that create the conditions of possibility for new knowledge and scholarship in this space. Through this engagement we also consider the *sociotechnical* research infrastructure needed to support the development of expertise, training and access models, as well as how these infrastructures in turn shape the nature of the research process itself.

To this end, we develop three conceptual devices (*orientating*, *auditing* and *constructing*) to frame a set of common activities that researchers must grapple with when first engaging with national web archives. Broadly, these overlapping concepts encompass practices (and associated challenges) related to accounting for the various idiosyncrasies of web archives; situating the archival/data sources within one’s own research paradigm and praxis; and confronting the opportunities and constraints of institutions and collections as research infrastructures. Rather than a linear workflow or fixed set of practices, these concepts necessarily overlap in ways that reflect the complex, iterative and often exploratory processes involved in the development of web archival research projects. Below, we outline each concept, and compare and contrast our own experiences at the UKWA and the Netarchive, with a view towards identifying how our research was shaped by the situated arrangements of each of these national web archives.

### Orientating

- ***orientating*** *: finding one’s position in relation to unfamiliar surroundings; tailoring or adapting to specified circumstances*

*Orientating* to the web archive includes engaging with web archives as new ontological devices for historical research; unpicking the often complex legal constraints of access; and embracing new ways of knowing data and infrastructure. Beyond the occasional use of the Internet Archive’s Wayback Machine, we acknowledge that most researchers attempting to use a national web archive will likely have never encountered a web archive or used web archival data before. Here we recognise that even coming to grips with *what a web archive is* can be challenging. As Ankerson (2015) describes, the process of adapting to the web archive as a source for research can be a daunting task for scholars unfamiliar with the idiosyncrasies of this ‘new type of historical document’ (Brügger, 2008). Researchers must grapple with concepts that have been transposed from the domain of document and paper-based archives,^4^ as well as terminology particular to web archiving (e.g. crawlers, captures, seeds). Distinguished from other ‘born digital’ material, Brügger (2016) characterises web archives as ‘re-born digital’ objects that are fundamentally shaped by the processes undertaken during their collection and preservation. Understanding the different approaches to web archiving collection activities (e.g. snapshots) can have important effects on the interpretation of archival search results and subsequent analyses - processes which we further discuss below in reference to *auditing* a web archive collection.

Researchers must also unpick the situated legal conditions of collection and access that both enable and constrain collection development and research. This particular aspect of orientating can be especially challenging in the face of different national legislation frameworks pertaining to copyright, data protection and digital publication rights online. In both cases, while we were both aware of access limitations prior to beginning these studies and fieldwork, neither of us were fully attuned to the ways that both UK legal deposit and Danish data protection laws intricately impacted every aspect of research. For the UKWA, access restrictions to the national web archive collections are divided between those web resources collected under selective, permissions-based crawling (the ‘Open UKWA’) and those harvested under the UK legal deposit scheme (the ‘Legal Deposit UKWA’). Due to the legal constraints surrounding the distribution of legal deposit publications, access to the Legal Deposit UKWA is restricted to on-site computer terminals in reading rooms that use a custom tool for searching and retrieving archived websites.^5^ In 2018, a new UKWA online search interface was released in an attempt to bring together the materials collected under legal deposit alongside the Open UKWA. The latest search interface uses faceted and full-text search, however off-site availability is still dictated by the express permission of rights holders. In this context, researchers must navigate the ways that the legislative environment places restrictions on the accessibility of resources harvested through legal deposit where, unlike other forms of archival research (digital or otherwise), at no stage are researchers allowed to explore the underlying data.

The process of coming to grips with the UKWA as a source for research was greatly assisted through initial conversations with BL staff and researchers. These conversations were instructive in figuring out what types of questions could be asked using the UKWA, particularly given the various access constraints. Even with access to the UKWA team, it was difficult to operationalise research questions into workable digital methods - both of which are steeped in particular epistemologies and ways of understanding the archive as a source for research. In this way, ‘coming to know’ the suitability of research questions and methods was an iterative process of moving between the substantive research topic and the curatorial and technical expertise of the UKWA and BL.

In the Danish context, the Netarchive is not openly accessible to open browsing and users must apply for access connected to specific research purposes. The type of access available depends on the nature of the research: access is provided by an on-site reading room at the library for personal use (or use by Masters students), while research users (including doctoral students) are provided with remote access by VPN connection. As part of the application, the researcher must also take on the responsibility of the legal role of ‘data controller’ and must consult with the Data Protection Agency if they intend to process sensitive personal data. While screenshots are possible in this context, data cannot be transferred or downloaded over the VPN to a researcher’s own machine. Like in the UKWA context, search and browsing of web archives is limited to search interfaces provided by the library, with no allowances for downloading data or access to WARC and derivative data formats.

Equally, orientating to web archives as sources for research forces scholars to critically engage with pre-conceived notions of the nature of archival or ‘big data’ research, and the ways in which these epistemological frames may or may not translate to the use of web archival resources in particular national web archival contexts. Working with the Netarchive required acclimatising to the scale of a national collection, compared to past experiences working with smaller Archive-It collections. During initial conversations with Royal Library staff and researchers, conceptual challenges arose surrounding the translation of our varied understandings of the nature of ‘a collection’. The scope of the Netarchive collection is closely aligned to their definition of ‘the Danish web’, which includes materials from the .dk top-level domain, as well as Danish materials beyond this domain determined by several criteria to be connected to Denmark. The library’s curators and researchers often assumed that Canada similarly has some form of holistic legal deposit for web materials or systematic harvesting by top level domain, and though there is some collecting at the federal government level by Library and Archives Canada, it has been fairly limited and inconsistent, and has been supplemented by significant collecting efforts by Canadian academic libraries (Milligan & Smyth, 2019; Wakaruk & Marks, 2019). In the absence of a comprehensive national web archive, researchers may study the Canadian archived web through smaller, disparate collections such as event- and subject-based Archive-It collections. In the Danish context, the Netarchive is large and almost impossible to view as a whole; to begin any kind of analysis, researchers must first develop strategies to select from hundreds of terabytes of data and pare down a subset that is relevant for their research. These different background experiences and contexts therefore influence how each curator or researcher defines and envisions a collection (and with what granularity) as a starting point for research.

### Auditing

- ***auditing*** *: inspecting, reviewing or assessing systematically; seeing the collection in the round, understanding boundaries*

*Auditing* the web archive includes engaging with the particularities of the collection and search interfaces of web archives; contextualising data by tracing a history of collection practices and curation decisions (to the extent they are knowable); and probing the limits and edges between data, collections and infrastructure. Access constraints, as well as current search interfaces make it difficult to see the collections ‘in the round’ or from a vantage point that gives a sense of where the boundaries of the archive lie. As Maemura et al. (2018, p.1225) discuss, questions of provenance in web archival collections research ‘broadly encompass what users need to know about how a collection was made’ in order to be confident in their analysis. Here, auditing is driven by a desire to ‘read against the archive’ (Zeitlyn, 2012), to assess (in)completeness and contextualise the archive by characterising what can be known about collection practices and their effects on the nature of national web archives. These activities aim to enable the formation of a more robust mental model of the collection(s), to capture histories of how archived artefacts come to be and identify gaps that impact potential use.

Recent interface developments elsewhere have experimented with presenting some of this contextual information about the collection process while browsing archived web pages - for example, the Internet Archive’s ‘about this capture’ interface (Graham, 2017) and Rhizome’s *Periphery* tool (Brucker, 2020). In the case of the UKWA and Netarchive, these types of interactions or metadata were less obvious or transparent through publicly available means.^6^

**Fig. 1:**
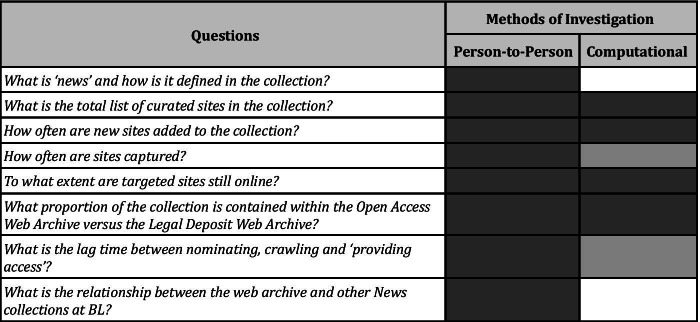
A selection of questions that drove ‘auditing’ the UKWA News Collection and the methods used to investigate the collection

Accordingly, conversations with subject curators and other researchers proved useful for both cases in assessing the impact of curation decisions on the types of questions that might be asked of the archives. As the UKWA contains collections that are curated by subject specialists, there was demonstrable value in situating the web archives within the wider News Collection, one which spans catalogues, mediums and subject areas at the BL. Figure 1 illustrates some questions that directed these auditing activities, as well as the methods used to explore the collection (both through person-to-person interactions with curators and by using computational means and the ACT tool, described below). Questions were aimed at unveiling complex issues surrounding the relationship between how news is defined and therefore collected and curated across mediums of production - including the Newspapers, Web news and Broadcast news (television and radio) collections. It was learned that each of these News collections often have different curators, collection models, discovery tools, catalogues, methods of access and lag-times between when they are collected and made available for use. In particular, a better understanding of the development of the Web news collection was achieved, including the subjective trade-offs between consistency and frequency of archival captures, sources used for creating new seeds, emergent issues of the ephemerality and sustainability of local Web news production, and the general resource (time, budgetary) constraints of web archiving. Further insights were also gained through discussion with the UKWA team, particularly around the nature of the domain scale crawling activities, the management of curated collections and the mechanisms put in place to restrict the harvesting of web sites to those that fall within the UK top-level domain or are otherwise verified as UK-based ‘publications’.

**Fig. 2:**
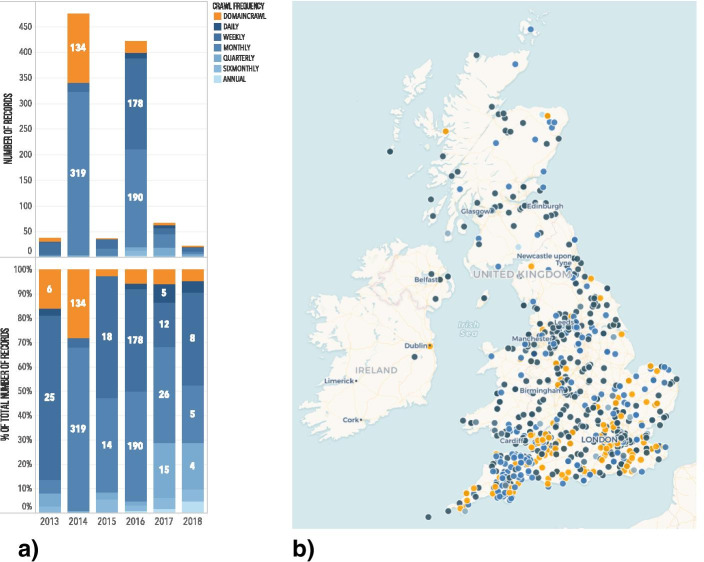
The crawl frequency of the UKWA Hyperlocal News collection targets between 2013 and 2018, presented as: **a** the total number of records and percentage of the total across crawl frequencies and **b** crawl frequencies mapped using local place names embedded within the target domain names

In addition to conversations with curators, the W3ACT - or *Annotation Curation Tool* (ACT) - was also used as a way to further contextualise the Hyperlocal News collection (in particular) and source links to construct a corpus to work with. In preparation for the Non-Print Legal Deposit regulations, ACT was developed by the UKWA in order to allow subject specialists and curators the ability to develop collections, add seeds and metadata, determine crawl frequencies, perform quality assurance and request and manage permissions from rights holders (Bingham & Byrne, 2016). Using basic curation data extracted from ACT and other attributes ascertained through the use of scripting, the Hyperlocal News collection was modelled to build up a picture of the collection’s curation over time. These auditing exercises provided clues to questions pertaining to the scale and frequency of captures (Fig. 2a), current website availability online and in this case, how the collection mapped geospatially (using place names extracted from the web domains themselves, see Fig. 2b). Mapping this collection provided a reminder that the UK domain (and legal deposit remit) also extends to UK Overseas Territories. The map makes it readily apparent that for example, there is only one site each for Gibraltar and Bermuda, and collection coverage in places like Northwest England and Scotland is notably sparse. Although this raises further questions about whether or not this is a feature of the local news environment in these places (a substantive question in its own right), auditing the Hyperlocal News collection in this way has clear implications for not only understanding the spatial distribution of the archives, but also the types of questions that might be asked of this collection.

In the case of the Netarchive, this sort of detailed investigation or modelling was not performed in order to answer a specific research question.^7^ However, it was similarly necessary to learn about the tools and processes used by curators to develop the collection. Here, auditing began with developing a better understanding of the Netarchive’s scope - characterised in terms of three core harvesting strategies (*Event*, *Selective* and *Broad*) that were revealed through engaging curators, other researchers and reviewing legal documents and collection policies. It was learned that *Event* harvests have a relatively small number of seeds and are crawled over a short time frame spanning several months. *Selective* harvesting focuses on a small set of sites, crawled at a high frequency (either weekly or daily), whereas *Broad* harvests capture all Danish web domains (over 1 million) four times a year, and therefore result in the greatest volume of material generated by the three strategies. But even with these descriptions, it still took further time and research to fully understand that these strategies, in effect, resulted in several different sub-collections, each with different scoping parameters.

**Fig. 3:**
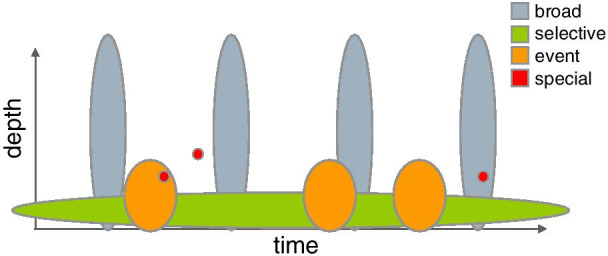
The ‘Bubble Diagram’ depicting the different harvest strategies at the Netarchive

In many early discussions curators mentioned or made reference to the ‘bubble diagram’ that visually summarises these harvesting strategies. For curators and other researchers more familiar with the Netarchive collection, the *Bubble Diagram* was a quick shorthand to express the varying depth and temporality of each harvesting strategy, plotting the crawl depth against the frequency of captures over time for each (Fig. 3). As the diagram has become central to how collecting is envisioned, changes to collection policies are also reflected in updates to the diagram, resulting in nuanced adjustments to the relative sizes of the bubbles. However, as a newcomer to the collection, fully understanding and reading the nuances of this diagram (and its effects) proves more difficult.

**Fig. 4:**
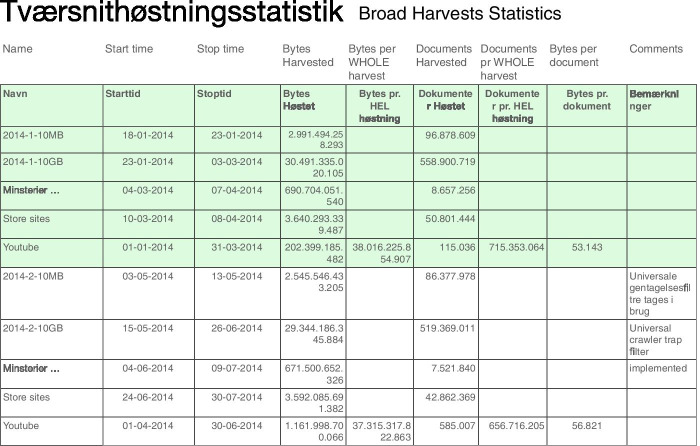
Broad harvest statistics summarising quantities of data and timing of captures

Though initially serving as a useful aid, the diagram also became a sort of barrier to deeper conversations about curation decisions. For example, while Broad harvests are represented diagrammatically as four individual captures throughout the year, the more detailed view described by curators addressed the hundreds of crawl jobs that generate this archived data, with different data limits applied to different seeds. Some of these limits are determined automatically by the harvesting system, but for certain domains, curators analyse the volume of data captured for each domain in the past and may adjust limits accordingly. The results of these analyses determine when and how a domain is crawled - with some crawls running simultaneously and others following a consecutive, stepwise process. Curators consider all of these varied, heterogeneous data together as contributing to a single Broad harvest - each of which is represented more simply as a contiguous bubble with a high dimension of ‘depth’ in the diagram. Through its abstract form, the diagram reduces the complex reality of harvesting (with many starts, stops and anomalies in the crawling process) to a relatively smooth and cohesive representation. More specific crawl configurations, limits and data quantities are found in other documentation like the public website and internal curator Wiki that records harvesting policies, detailed descriptions of each strategy and statistics for data captured in each crawl. An excerpt from this internal documentation (Fig. 4) illustrates a more fine-grained view of the data: the five lines highlighted in green represent data captures for the first Broad harvest in 2014, showing the different timing, limits applied and overall quantities of data harvested. In this case, auditing requires moving beyond the diagram, and looking more closely at finer-grain details through review of this documentation and discussions with curators.

### Constructing

- ***constructing*** *: building or making something, to form an idea or theory by bringing together conceptual elements*

*Constructing* encompasses activities surrounding the creation of a subset of data to work with through more focused analyses. This includes negotiating and navigating the technical infrastructure to access diverse and varied forms of data; selecting and aggregating data from sources across ‘collections’; and iteratively revisiting the possibilities of particular research methods given data availability. Constructing a corpus extends beyond conventional online browsing of individual ‘documents’ or archived web pages through interfaces such as Open Wayback. Although Winters (2019) discusses how this form of qualitative research has been normalised due to legal deposit access restrictions, here we also include the needs of researchers with qualitative research aims and methods (e.g. thematic or content analysis) to be able to source and extract their own corpus with which to perform their analysis through computational means. Elsewhere Ben-David and Huurdeman (2014) have argued that the arrival of search-enabled interfaces powered by full-text indexing permits a transition from the document as the unit of analysis to discovery mechanisms that facilitate corpus and collection-based analyses in web archives. Here full-text search is seen as a major asset that not all web archives have the capacity to provide, or indeed keep updated as the collection changes, especially at the national domain scale.

The corpus therefore represents a stable dataset with which to perform analysis, one that can be queried with custom scripting and applications, as well as enable researchers to clearly define and demarcate the boundaries of their research enquiry. Motivations for creating a corpus are also potentially reflective of other concerns around the nature of pre-existing ‘special collections’ - where these archives may not directly map to the substantive or methodological needs of particular research agendas. Here, tools and interfaces for facilitating the re-imagining or drawing together of collections data are needed, where researchers are allowed to aggregate archived sources that may span across collections, harvesting strategies or institutional mandates for collection.

Elsewhere, approaches to sourcing link-lists for corpus-building are often reliant on a mix of methods that can include using known seed lists for particular topics and domains (Ben-David, 2016), using iterative or ‘snowball’ techniques for collecting URIs from networked web pages and sites like Wikipedia (Ben-David, 2016; Ben-David and Amram, 2018), or extracting links from other discrete datasets such as the links shared within a particular social media archive. However, there is an obvious tension that emerges between this desire to stabilise or collate resources and the legal frameworks that govern data access in the context of national web archives. For both the UKWA and the Netarchive, self-guided exploratory work using WARC data to examine networks of links, keyword search queries, named entities extraction, etc. is restricted based on legal deposit regulations in the former, and data protection laws for the latter.^8^

In the study of the Netarchive through this three-month fieldwork exercise, the construction of a corpus was out of scope due to the limits imposed by this legal context. Access and movement of data from the library’s servers is prohibited, preventing any data analysis and exploration on an individual researcher’s computer. However, the library has recently developed new mechanisms to support large-scale data analysis, through projects run on their National Cultural Heritage Cluster. A research project team may apply to work with this high-performance computing infrastructure to study the Netarchive collection, and successful projects received support and guidance from a dedicated data engineer. To adhere to data protection laws, the library controls which data may move from the library’s storage servers to the cluster. Additionally, using this high-performance computing infrastructure means that performing an analysis is not just a matter of plugging in standard code - more commonly used code statements must be translated to a language compatible with distributed processors (for example, adjusting syntax for PySpark as opposed to Python).^9^ In this context, a study that involves large-scale data analysis means envisioning a long-term, team-based endeavour with significant planning (rather than an ad-hoc or one-off data exploration initiated by an individual researcher).

In the UKWA, the creation of a corpus was driven by the substantive nature of the case study research, which required the collation of archived news stories that mentioned ‘immigration’ or ‘migration’ within the Web News and Hyperlocal News collections. In an ideal scenario, an additional search on the publication dates would have then returned a corpus of news stories mentioning these keywords with which to further explore. However, whilst using the equivalent of keyword and date searches can be seen as a common approach to creating datasets of this nature, a number of challenges made collating a corpus difficult under the constraints of both the search interfaces provided by the UKWA and the legal framework governing access to archived News sites collected under legal deposit. During this research, the main UKWA search interface and software was under extensive development which presented its own problems for reliably searching the UKWA, however, there were more fundamental issues at play. As one example, searching by date is limited to the *date the site was crawled*, rather than the *date of publication*. Since the process of indexing relies on the standardized date and time data recorded by the crawler, addressing the messier (and often absent) information on the publication date of each item in the collection would be a resource intensive exercise to complete and maintain at scale, especially over time. However, this means that the UKWA search interface does not allow for filtering or time-bounding search results within a particular publication date, which is particularly necessary for the study of news coverage over time. Furthermore, without being able to download the results - or even a summary report of the URLs returned in a keyword search - additional self-guided exploratory work using other existing analysis tools is rendered impossible.

In an effort to circumvent these constraints, some proof of concept work was undertaken using remote access to the Solr index that powers the current UKWA search user interface. Using the Solr index enabled a focus on the Hyperlocal News collection - a ‘curated collection’ not currently made available through the main UKWA search facets. With the help of team members who provided assistance with access issues and tips on Solr syntax, rudimentary queries were performed to establish how many ‘documents’ (in Solr terms) in the Hyperlocal News collection included the keywords relevant to the case study on immigration discourse in the UK. The selection of this subset from the Hyperlocal News collection was intended to allow for a comparison with the findings from auditing the collection using the ACT tool (discussed earlier), with the aim of mapping these sites geospatially. Comparing the Solr search results with the ACT curated URLs revealed a number of ‘archival artefacts’ (Ben-David & Huurdeman, 2014, p.104) including crawl oddities in domain coverage (e.g. non-news sites) and significant time delays between when sites are crawled, indexed and made available through full-text search.^10^ This ultimately raised questions about the nature of ‘collections’ and their fit for purpose for these sorts of digital research enquiries - including questions about the boundaries of curated collections (when crawls stray from the target URIs) and the resources required to make them available.

## Discussion and analysis

The above examples detail some of the ways that researchers must grapple with web archives as sources for research before data analysis or indeed, any practical or material readings of ‘data’ can proceed. While each of our projects focused on a national web archives collection, the specific methods applied (and subsequent resolution of challenges or roadblocks) are not easily transferable across sites of research, due at least in part to the different legal frameworks that govern these web archives. However, both projects necessitated a combination of expertise, dialogue, creativity and flexibility in order to engage with these research infrastructures and the everyday human and technological interventions required to facilitate access to the UKWA and Netarchive. Building on the work of ‘scholarly primitives’, we contribute a conceptual framework designed to capture and illuminate the researcher activities (and associated challenges) at the earliest stages of this form of scholarship.

First, *orientating* is required for researchers to become acquainted with a collection and broadly consider the conditions surrounding a collection that impact its use. This includes becoming familiar with specific limits and constraints, legal governance frameworks, collection mandates, as well as configurations (i.e. of sub-collections) and terminology used for specific collections. Second, we identified a number of ways that *auditing* a collection must be performed prior to data selection and analysis. This includes activities to explore and model the collection through readings of metadata and supporting documentation that provides evidence of the everyday infrastructural maintenance and labour involved in web archiving. Addressing these socio-material histories of web archives ultimately serves to develop an understanding of the epistemic practices that drive collection activities and shape the nature of the archives. And finally, we discussed the ways that *constructing* a corpus in the context of national web archives is both a significant challenge and a foundational activity for enabling archival research and analysis. The activities and processes at this stage include working within the affordances and path dependencies of tools and technical standards, as well as negotiating the various interfaces through which web archives are accessed by researchers. While few accounts of methodologies address the decision-making at this early stage prior to data analysis, we emphasise the laborious and epistemologically grounded nature of this work, and additionally highlight how the infrastructure and researcher are both active agents in this process, in effect co-constructing the research in practice.

A core contribution of presenting and analysing this work through the conceptual framework developed here is a greater recognition of the significant time and energy required on the part of researchers to begin engaging with web archives. Future projects must acknowledge and allow time for researchers to grapple with tensions between expectations of access to these digital resources - programmatic or otherwise - and the legal and regulatory framework under which access is determined. Acclimatising to the different modes of describing, accessing and analysing ‘re-born’ digital archives is one of the biggest challenges of using national web archives. As is also observed by others (Winters, 2019; Milligan, 2015), in many ways web archives in this context are *antithetical* to research - they are composed of born-digital, previously online materials that are rendered largely inaccessible online (in archival format) due to the very mechanisms that enable their collection (e.g. legal deposit). Our experiences point to the need for creative interventions and workarounds for enabling and adapting pre-existing digital research methodologies to the national web archive environment. In consultation with library staff, we argue that these archival engagements therefore require dialogue, time, subject and technical expertise and often, the optimism to ‘stay with the trouble’ (Haraway, 2016) in the face of major obstacles to use. Here, the focus on harvesting activities must be met with greater collective investment into understanding how to communicate the (varied and complex) affordances of these collections to researchers.

Additionally, our work points to the value of engaging with the curatorial infrastructure that surrounds the creation of web archives in the national web context and the importance of curators’ involvement in discussions at these early stages of research. Opportunities for exploring the UKWA ACT tool and curators’ internal documentation at the Netarchive potentially offer additional capacity for researchers to engage with available metadata about a collection in order to understand contextual elements which have bearing on use. Although not used in these studies, there is also potential for engaging crawl logs to explore domain scale harvests. In both the UK and Danish legal contexts, there are fewer limitations to sharing metadata about crawled web archival data, therefore enabling researchers to access an additional form of derivative web archival data to facilitate these types of early engagement activities. However, these explorations also invite us to consider the relationship between records and the surrogate material objects they represent. In the case of the UKWA, ACT records (and associated metadata) are sociotechnical constructs, ‘acts of reduction’ and representation (Schnapp, 2013), so to treat them as replicates for the web objects that we ultimately want to study is not without problems. On the other hand, these forms of data are constructs that speak to the affordances of web archives, the ways in which they are being managed and maintained and the potential implications for their use as sources for research.

Finally, we close with a note of caution, to address how each access interface and point of entry to the web archive serves to frame these resources in specific ways, constraining the types of questions that may be asked. This observation aligns with Ben-David & Huurdeman’s (2014, p.94) discussion of working with the Wayback Machine and the ‘embedded temporalities’ of access interfaces for web archives - where imagined use cases at the time of development are privileged, often at the expense of enabling future research practices. In our studies we initially expected to access WARC data - where the scale and units of analysis would move interchangeably between single URLs (*document-centric* views) and the wider ‘collection(s)’ (*data-centric* views). However, working within the limitations of access, we relied on other representations and views into web archives at different junctures, including the use of Solr search indices and query interfaces, faceted and free text search interfaces under development, curation tools (which provided different seed- and collection-centric views), and numerous ad-hoc and incomplete data representations (JSON, CSV/XSL and PNG screenshots). Our engagements also spawned reflections on the tacit approaches to interacting with different interfaces and the ways that researchers need space in the research process to fiddle, explore and hover. Here, we are reminded of the experience of Milligan (2015) in the UKWA, and their comment that as a researcher they needed to see the source code as part of their research practice.^11^ We consider how this is indicative of the wider, subtle and under-documented digital practices associated with web archival scholarship where, as researchers, we want to ‘check under the hood’ and view the inner workings of the archive; to contextualise through opening, seeing and sense-making practices that are often made difficult (or near impossible) by the access restrictions presented in national web archives.

In short, the gaps and interruptions in our experiences are also equally revealing of the infrastructure and contingencies of web archives. Here we propose that these interfaces may be viewed as sites of web archival *data friction* (Edwards, 2013) that often delays and obstructs the research process in web archives. As Edwards (2013, p.83-85) details, the data friction metaphor represents the socio-material resistance that ‘occurs at the interfaces between objects and surfaces’ and impedes the use, movement or transfer of data. Web archival data are therefore recognised as both *process* and *products* of labour by a spectrum of creators and possible users that have an influence on the ways this data may be used. Data friction emphasises the importance of acknowledging the ‘materialities, productivities and mediating capacities’ of these types of digital data and the apparatuses which enable/produce data collection and use (Ruppert et al., 2013, p.24–25). Researchers wishing to use these archives therefore must attend to the material contingencies of the infrastructures that shape and produce emergent sources of data, and these projects highlight how a researcher might go about exploring and accounting for the processes that underpin web archival collections. By reframing or reconfiguring these frictions as sources of knowledge (as opposed to tacit gaps or dead ends), we believe they hold potential to aid and inform researchers of the underlying sociotechnical infrastructures supporting collections and the ways they may be used to further the research process.

## Conclusion

There has been an intensive focus within the field on supporting the development of new tools and interfaces for using web archives. In the context of two national web archives, we explored the challenges researchers face before even beginning to engage with tools and interfaces to analyse data directly. We characterise this set of activities through a conceptual framework that attends to the entanglement of epistemic practices (both on the part of researchers and national web archive curators/developers), legal restrictions and the software used to enable harvesting, curation and user access. Whereas the existence and promotion of research programmes, ‘datathons’ and workshops have worked towards providing introductory or general use tools for analysing web archives, we propose that enrolling DH and digital social science scholars in web archival research via this framework facilitates equally necessary knowledge about how to engage with curatorial infrastructure, what questions to ask and what resources beyond a collection’s ‘data’ may structure the research design process.

We additionally envision that a focus on these early research activities of orientating, auditing and constructing, can promote greater investment and resources devoted to negotiating the (potentially) broad gap between a research question and the answers provided by available data and tools. It is evident in our initial explorations that interfaces guide researchers (at least implicitly) to structure an analysis around that which is more readily available through default values in the data (i.e. counts by domain), as opposed to the inherently ambiguous steps in arriving at something closer to their research aims. Although we recognise the resource constraints under which most web archiving programmes operate, the value of curators engaging with researchers directly at the earliest stages of research should not be underestimated. We propose that future work should further consider how to identify and trace the sources and effects of data frictions, with a view towards developing collaborative environments for researchers to work with the sociotechnical infrastructure of national web archives. In this way, ‘go fish’ becomes less an impossible demand, but an act of optimism - a prompt to explore the space between the ‘future promise’ of web archives and the contemporary challenges they invoke for researcher use.

## Footnotes

1 https://github.com/helgeho/ArchiveSpark

2 https://archivesunleashed.org/aut/

3 The legal deposit libraries include: the British Library, the National Library of Wales, the National Library of Scotland, University of Oxford Bodleian Libraries, Cambridge University Library and Trinity College Library, Dublin. Prior to the establishment of the UKWA, the British Library was involved in ‘selective’ web archiving collection activities dating back to 2001, with the *UK Domain Project* (later renamed the *Britain on the Web* project) (Day, 2003).

4 See Baker (2013) and Ben-David and Huurdeman (2014) for discussions of the potential trappings of page-based nomenclature and methods of access in the preservation of web resources. In the case of our studies discussed here, we have both spent several years engaging with web archiving and were familiar with fundamental concepts prior to beginning fieldwork. In this regard, our experiences will almost certainly not be representative of what a ‘typical researcher’ might first encounter, yet we both experienced challenges in attempting to use these collections.

5 Milligan (2015) discusses the experience of using the Legal Deposit UKWA tool, where the document-style interface and restrictions placed on simultaneous viewing of archived ‘publications’ render typical research practices like checking the underlying source code of archived pages, taking screenshots and even copy/paste unworkable.

6 The Netarchive has since implemented changes with their SolrWayback interface (https://github.com/netarchivesuite/solrwayback) which addresses some aspects of provenance like making note of temporal inconsistencies between resources presented on a page.

7 Though not the focus of an extensive analysis in this case, the Netarchive’s curators also document their selection decisions through their own tools (providing collection development support like the ACT does for the BL), most notable with an internal curator Wiki and Jira issue tracking.

8 In the case of the UKWA, all previous large-scale research has been therefore enabled by the availability of the UK Web Domain dataset (also known as the ‘JISC archive’), which contains all sites crawled under the .uk domain by the Internet Archive between 1996 and 2010 - and later expanded to include data through April 2013 (JISC and the Internet Archive, 2013). However, as this case study was focused on the news coverage of a particular UK policy change that occurred in late 2013, the JISC archive did not provide the means to explore this specific research agenda - highlighting one of the limitations of relying on this dataset to access .uk domain data at scale over time.

9 Details on the cluster’s pilot project and the process of establishing a corpus from Broad harvests are described in the chapter by Brügger, Laursen and Nielsen in Brügger & Laursen, 2019.

10 At the time of the study (2018), the index was reportedly three years behind domain crawling activities - meaning the UKWA full-text search only included sites archived up to 2015.

11 Also, see discussion in Winters (2019).
